# Supplementary figures and images for: Enhanced Outer Membrane Vesicle Production in Escherichia coli: From Metabolic Network Model to Designed Strain Lipidomic Profile
Source: Int J Mol Sci. 2025 Jul 13;26(14):6714. doi: 10.3390/ijms26146714 (PMC12295046; doi:10.3390/ijms26146714)

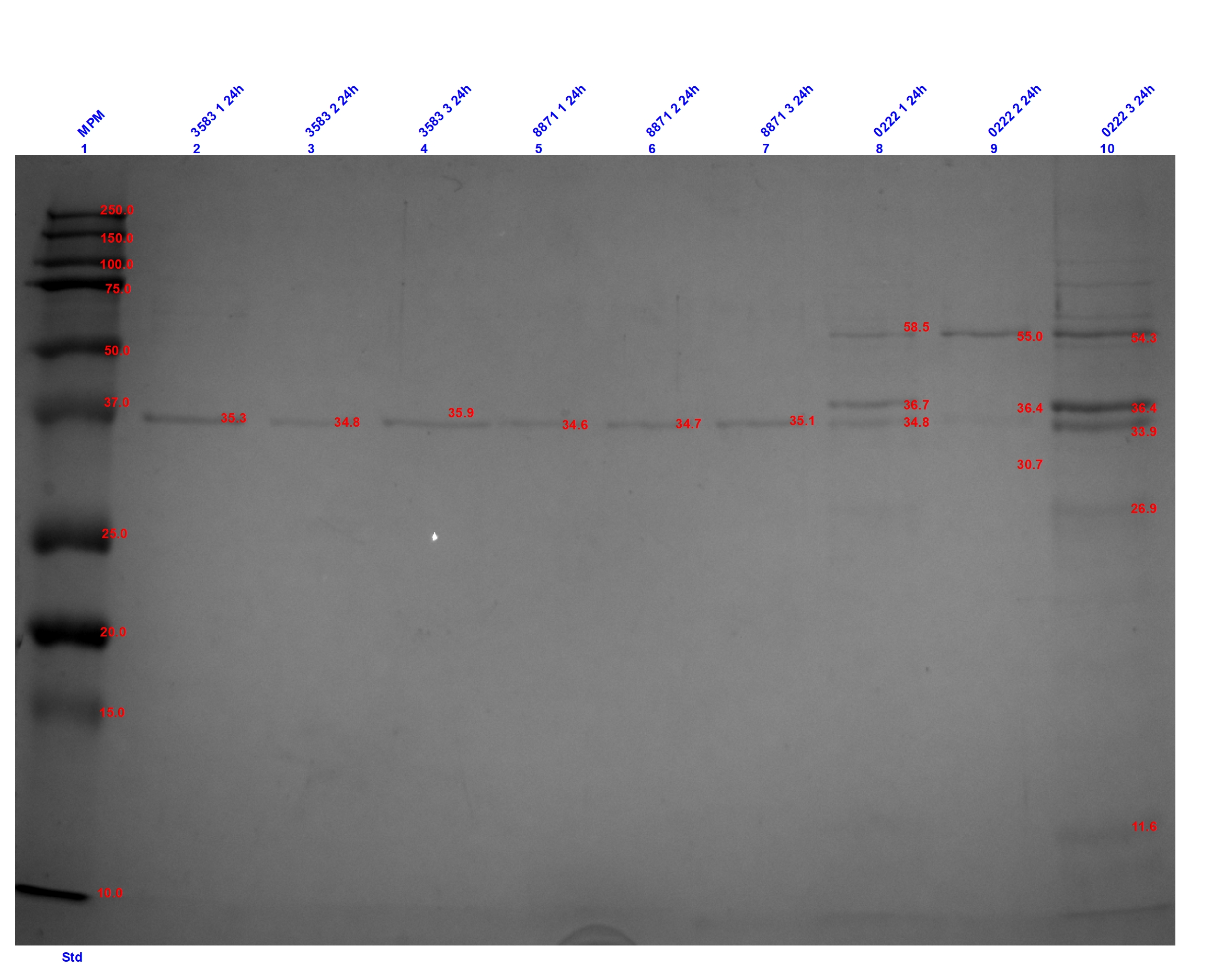

Supplement: Supplementary file 1 [file ijms-26-06714-s001.zip › File S1. SDS-PAGE .jpg]
